# Genistein Inhibits Prostate Cancer Cell Growth by Targeting miR-34a and Oncogenic HOTAIR

**DOI:** 10.1371/journal.pone.0070372

**Published:** 2013-08-01

**Authors:** Takeshi Chiyomaru, Soichiro Yamamura, Shinichiro Fukuhara, Hirofumi Yoshino, Takashi Kinoshita, Shahana Majid, Sharanjot Saini, Inik Chang, Yuichiro Tanaka, Hideki Enokida, Naohiko Seki, Masayuki Nakagawa, Rajvir Dahiya

**Affiliations:** 1 Department of Urology, San Francisco Veterans Affairs Medical Center and University of California San Francisco, San Francisco, California, United States of America; 2 Department of Urology, Graduate School of Medical and Dental Sciences, Kagoshima University, Kagoshima, Japan; 3 Department of Functional Genomics, Graduate School of Medicine, Chiba University, Chiba, Japan; Wayne State University, United States of America

## Abstract

**Objective:**

Genistein is a soy isoflavone that has antitumor activity both in vitro and in vivo. It has been shown that genistein inhibits many type of cancers including prostate cancer (PCa) by regulating several cell signaling pathways and microRNAs (miRNAs). Recent studies suggest that the long non-coding RNAs (lncRNAs) are also involved in many cellular processes. At present there are no reports about the relationship between gensitein, miRNAs and lncRNAs. In this study, we focused on miRNAs, lncRNA that are regulated by genistein and investigated their functional role in PCa.

**Method:**

Microarray (SurePrint G3 Human GE 8×60K) was used for expression profiling of genistein treated and control PCa cells (PC3 and DU145). Functional assay (cell proliferation, migration, invasion, apoptosis and cell cycle assays) were performed with the PCa cell lines, PC3 and DU145. Both in vitro and in vivo (nude mouse) models were used for growth assays. Luciferase reporter assays were used for binding of miR-34a to HOTAIR.

**Results:**

LncRNA profiling showed that HOTAIR was highly regulated by genistein and its expression was higher in castration-resistant PCa cell lines than in normal prostate cells. Knockdown (siRNA) of HOTAIR decreased PCa cell proliferation, migration and invasion and induced apoptosis and cell cycle arrest. miR-34a was also up-regulated by genistein and may directly target HOTAIR in both PC3 and DU145 PCa cells.

**Conclusions:**

Our results indicated that genistein inhibited PCa cell growth through down-regulation of oncogenic HOTAIR that is also targeted by tumor suppressor miR-34a. These findings enhance understanding of how genistein regulates lncRNA HOTAIR and miR-34a in PCa.

## Introduction

Genistein is a dietary soy isoflavone. Its structure is similar to that of human 17-β-estradiol causing estrogenic and/or antiestrogenic effects [1]. Genistein is also a protein tyrosine kinase inhibitor [2] and has antitumor effects in vitro and in vivo. It has been shown that genistein inhibits many type of cancers including prostate cancer (PCa) [3], [4] through regulation of several cell signaling pathways such as the Wnt, Akt and JAK/STAT pathways [5]–[8].

Recent evidence suggests that non-coding RNAs (ncRNAs) are involved in many cellular processes. microRNAs (miRNAs), class of small ncRNAs about 22 nucleotides in length, function as negative regulators of target mRNAs transcriptionally and post-transcriptionally [9]. It is known that miRNAs regulate up to two-thirds of the human genome [10] and play important roles in numerous biological processes including development, differentiation, proliferation, angiogenesis, metabolism and pluripotency [11], [12]. It has been reported that genistein increased expression of tumor suppressor miR-146a, causing inhibition of the EGFR and NF-kB pathway [13], [14]. miR-27a has been reported to be a oncogenic miRNA regulated by genistein and regulates VEGF signaling by targeting ZBTB10 [15], [16]. Our previous studies showed that genistein treatment significantly down-regulated the expression of oncogenic miR-151 which directly targets SOX17 and ARHGDIA [17]. SOX17 was reported to be a tumor suppressor gene that inhibits WNT/β-catenin signaling by targeting both β-catenin and T-cell factor (TCF)/lymphoid enhancer factor (LEF) proteins [18]–[20]. ARHGDIA negatively regulates the Rho family of GTPases (Rho, Rac, and Cdc42) [21] that are involved in the WNT signaling pathway [22]. We also found that genistein down-regulates the RAC1 and EP300 genes that are important regulators of VEGF-mediated angiogenesis [23], [24] and the EGFR gene by up-regulating miR-574-3p [25].

NcRNAs are divided into two major classes based on transcript size; small ncRNAs and long ncRNAs (lncRNAs). lncRNAs are in general defined as RNA genes larger than 200 nucleotides that have no protein coding potential. Large scale sequencing of cDNA libraries and next generation sequencing indicate that lncRNAs in mammals number in the tens of thousands. So far, only 126 human lncRNAs have been functionally annotated in lncRNA data base [26]. Thus there are no reports about the relationship between gensitein and lncRNAs.

The HOX transcript antisense RNA (HOTAIR) gene is located within the Homeobox C (HOXC) gene cluster on chromosome 12 and encodes s 2.2 kb lncRNA molecule. This gene is shuttled from chromosome 12 to chromosome 2 by a component of the Polycomb Repressive Complex 2 (PRC2) and represses transcription of homeobox D (HOXD) genes [27]. HOTAIR interacts with both PRC2 and lysine specific demethylase 1 (LSD1) complexes and couples histone H3 lysine 27 methylation and lysine 4 demethylation for epigenetic silencing of not only HOXD genes but also many other genes [28]. This gene is highly expressed in several cancers such as breast, colorectal, liver, pancreas, and laryngeal cancer [29]–[33]. High expression of HOTAIR in breast cancer is a predictor of metastasis and poor outcome [29]. HOTAIR is also thought to be a potential biomarker for the existence of lymph node metastasis in hepatocellular carcinoma [34]. HOTAIR is a negative prognostic factor and acts as an oncogene in pancreatic cancer both in vitro and in vivo [32]. Therefore in this study, we focused on the lncRNA HOTAIR that is regulated by genistein and investigated its functional role in PCa.

## Results

### Effect of Genistein Treatment on Proliferation, Apoptosis and Cell Cycle in PCa Cells

**Figure 1:**
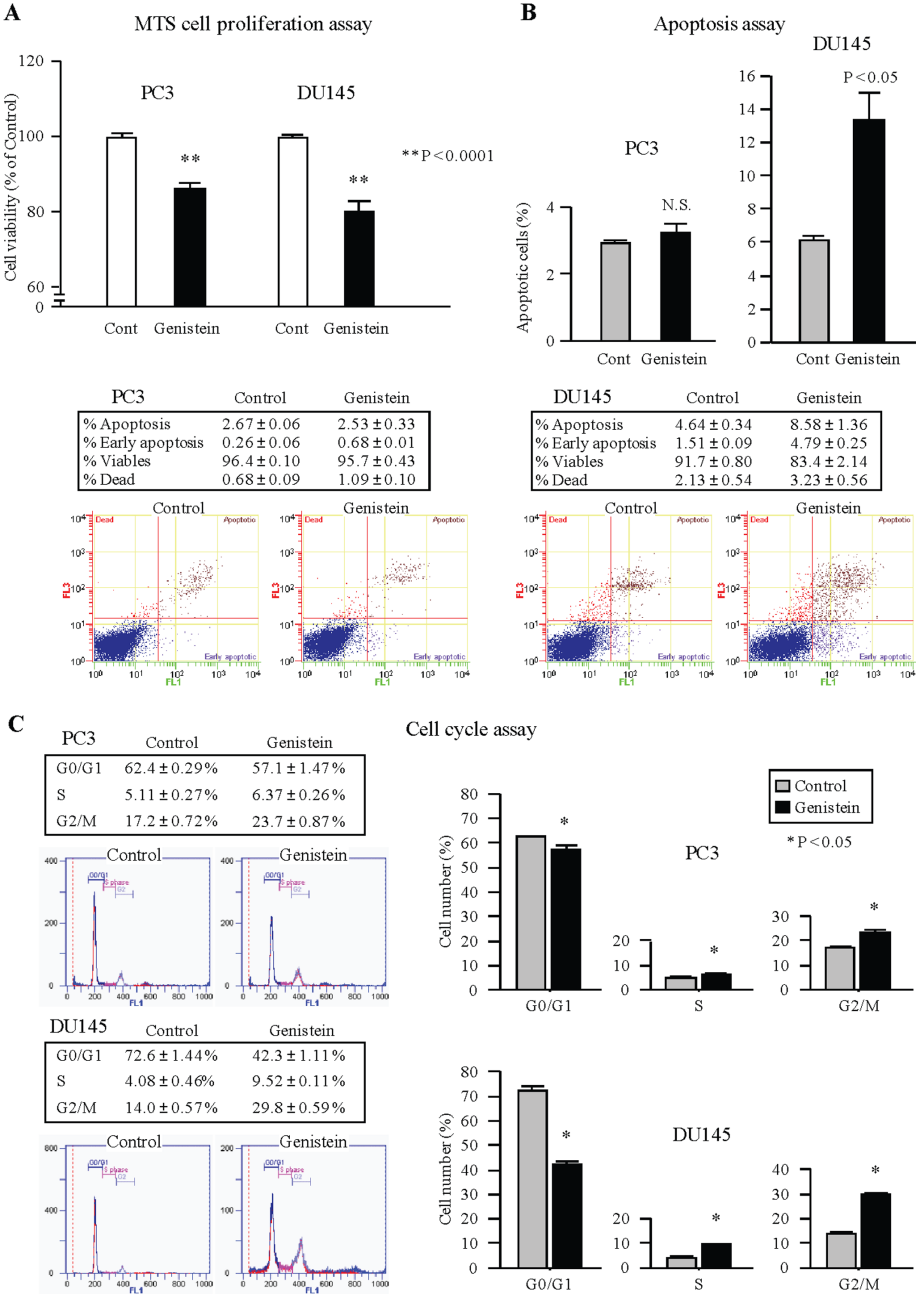
Effect of genistein treatment on PCa cells (PC3 and DU145). (A) Genistein significantly inhibits cell viability. Cell viability was analyzed by the MTS cell proliferation assay after 4 days treatment (25 µM). **, P<0.0001. *, P<0.01. Data are presented as the mean ± SE. (B) Apoptosis assay using flow cytometry. Representative quadrant figures of control and genistein (25 µM) treated cells in PC3 (left) and DU145 (right) cells. P<0.05. (C) The typical figures of cell cycle analysis of control, or genistein (25 µM) treated cells are shown. The bar chart shown on the right of each figures represents the percentage of the cells in G0/G1, S, or G2/M phase as indicated. *P<0.05.

To verify the tumor suppressive features of genistein, we conducted function analysis. The MTS assay showed that the cell proliferation was reduced by genistein treatment in both PC3 and DU145 cells (Fig. 1A). Apoptosis assay demonstrated that genistein significantly induced apoptosis of DU145 cells (Fig. 1B). However, no significant effect of apoptosis was observed in PC3 cells. Cell cycle assay demonstrated that treatment with genistein caused G2/M phase cell cycle arrest in both PC3 and DU145 cells (Fig. 1C).

### Identification of Target Genes and Molecular Pathways Regulated by Genistein in PCa

To identify genes and pathways targeted by genistein in PCa cells, we performed gene expression profiling using genistein and vehicle (control) treated PC3 and DU145 cells. The expression of 918 genes were significantly down-regulated in both PCa cell lines (average log2 ratio<–0.5, Table S1). These genes were assigned Kyoto Encyclopedia of Genes and Genomes (KEGG) annotations using singular enrichment analysis of GeneCodis. Significantly enriched pathways in KEGG were identified such as ‘Pathways in cancer’, ‘Cell cycle’, ‘Regulation of actin cytoskeleton’ and ‘Jak-STAT signaling pathway’ (P<0.05, Table S2). We focused on the KEGG ‘Pathway in cancer’ and the genes in this pathway are highlighted in the KEGG map (Fig. S1).

To determine gene expression in PCa clinical samples, we performed expression analyses for all candidate target genes involved in the ‘Pathways in cancer’ using microarray expression data, which were approved by the Gene Expression Omnibus (GEO). The expression levels of these genes were examined in 47 PCa and 47 normal samples, as shown in the heat map diagram in Fig. S2. Genes up-regulated in PCa are shown in red, down-regulated genes are shown in blue, while white bars indicate genes that are expressed at similar levels in both. From this analysis, several crucial gene targets were identified, including histone deacetylase 1 (HDAC1), protein inhibitor of activated STAT, 3 (PIAS3), tropomyosin 3 (TPM3), transcription factor 7-like 2 (T-cell specific, HMG-box) (TCF7L2), aryl-hydrocarbon receptor nuclear translocator 2 (ARNT2), CREB binding protein (CREBBP), junction plakoglobin (JUP), and v-akt murine thymoma viral oncogene homolog 2 (AKT2).

### Identification of lncRNAs Regulated by Genistein in PCa

**Figure 2:**
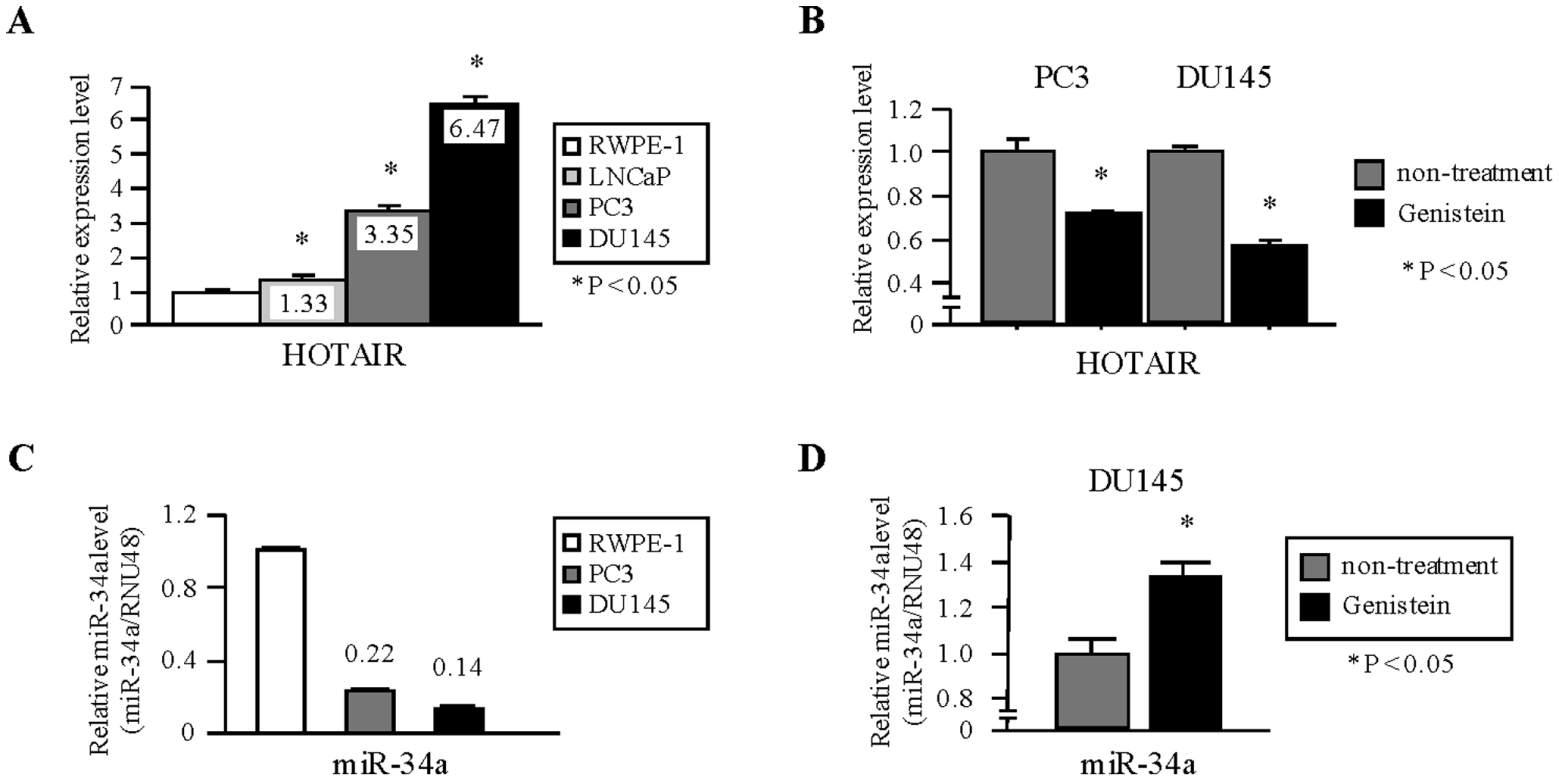
Effect of genistein treatment and expression of HOTAIR and miR-34a in PCa cells. (A) Expression of HOTAIR in PCa cell lines (LNCaP, PC3 and DU145) and normal prostate epithelial cells (RWPE-1). Real-time PCR showed that the expression levels of HOTAIR were up-regulated in castration-resistant PCa cell lines (PC3 and DU145). Data are presented as mean ± SE. *, P<0.05. (B) Expression levels of HOTAIR after treatment with genistein (25 µM). HOTAIR expression decreased by 30–40% in genistein treated cells compared with controls. HOTAIR expression was normalized to GAPDH. *, P<0.05. (C) Expression of miR-34a in PCa cell lines (PC3 and DU145) and normal prostate epithelial cells (RWPE-1). Real-time PCR showed that the expression levels of miR-34a were down-regulated in castration-resistant PCa cell lines (PC3 and DU145). miR-34a expression was normalized to RNU48. (D) Expression levels of miR-34a after treatment with genistein (25 µM) in DU145 cell. P<0.05.

The SurePrint G3 Human GE 8×60K microarray platform also includes probes for lincRNAs. To identify the lncRNAs regulated by genistein in PCa cells, we performed gene expression profiling. Five lncRNAs were down-regulated in both PCa cell lines (average log2 ratio<−1.0, Table 1) with lncRNA HOTAIR showing the largest effect. To determine relative expression levels of HOTAIR in prostate cells, we performed quantitative real-time PCR using untreated PCa cell lines and compared them with normal prostate epithelial cells (RWPE-1). We observed that HOTAIR expression was significantly up-regulated in castration-resistant PCa cell lines (PC3 and DU145) compared to RWPE-1 cells (PC3 3.35-fold, DU145 6.47-fold) (Fig. 2A). To confirm the microarray data, we performed TaqMan quantitative real-time PCR analysis and observed that HOTAIR expression was significantly down-regulated with genistein treatment (Fig. 2B). Thus we focused on HOTAIR because many investigators have reported that HOTAIR has an oncogenic function in several other cancers (breast, liver, pancreas, colorectal cancers and laryngeal squamous cell carcinoma) [29]–[33].

**Table 1:** Genistein down-regulated lncRNAs (less than −1.00-fold).

No.	Entrez gene ID	Symbol	Gene name	Log 2 ratio		
				PC3	DU145	Average
1	100124700	HOTAIR	HOX transcript antisense RNA	−1.92	−1.23	−1.58
2	100287628	LOC100287628	uncharacterized LOC100287628	−1.59	−1.35	−1.47
3	145474	LOC145474	uncharacterized LOC145474	−1.58	−1.31	−1.45
4	387097	C6orf147	chromosome 6 open reading frame 147	−1.56	−1.02	−1.29
5	100507165	LOC100507165	uncharacterized LOC100507165	−1.13	−1.05	−1.09

### Regulation of HOTAIR Expression in PCa Cell Lines by miR-34a

Recently, it was reported that some miRNAs regulate the expression of lncRNAs [35]–[37]. To search for potential miRNAs that regulate HOTAIR, we used miRcode algorithm. In several candidate miRNAs targeting HOTAIR, we focused on miR-34a since our lab has been previously reported functions as a tumor suppressor and is down-regulated in PCa tissues [38]. To determine the relative expression levels of miR-34a in prostate cells, we performed quantitative real-time PCR using PCa cell lines (PC3 and DU145) and compared them with normal prostate epithelial cells (RWPE-1). We observed that miR-34a expression was significantly down-regulated in castration-resistant PCa cell lines (PC3 and DU145) compared to RWPE-1 cells (PC3 0.22-fold, DU145 0.14-fold) (Fig. 2C). To search for the effect of genistein to miR-34a expression, we performed TaqMan quantitative real-time PCR analysis and observed that miR-34a expression was significantly up-regulated with genistein treatment in DU145 cells (1.36-fold) (Fig. 2D).

**Figure 3:**
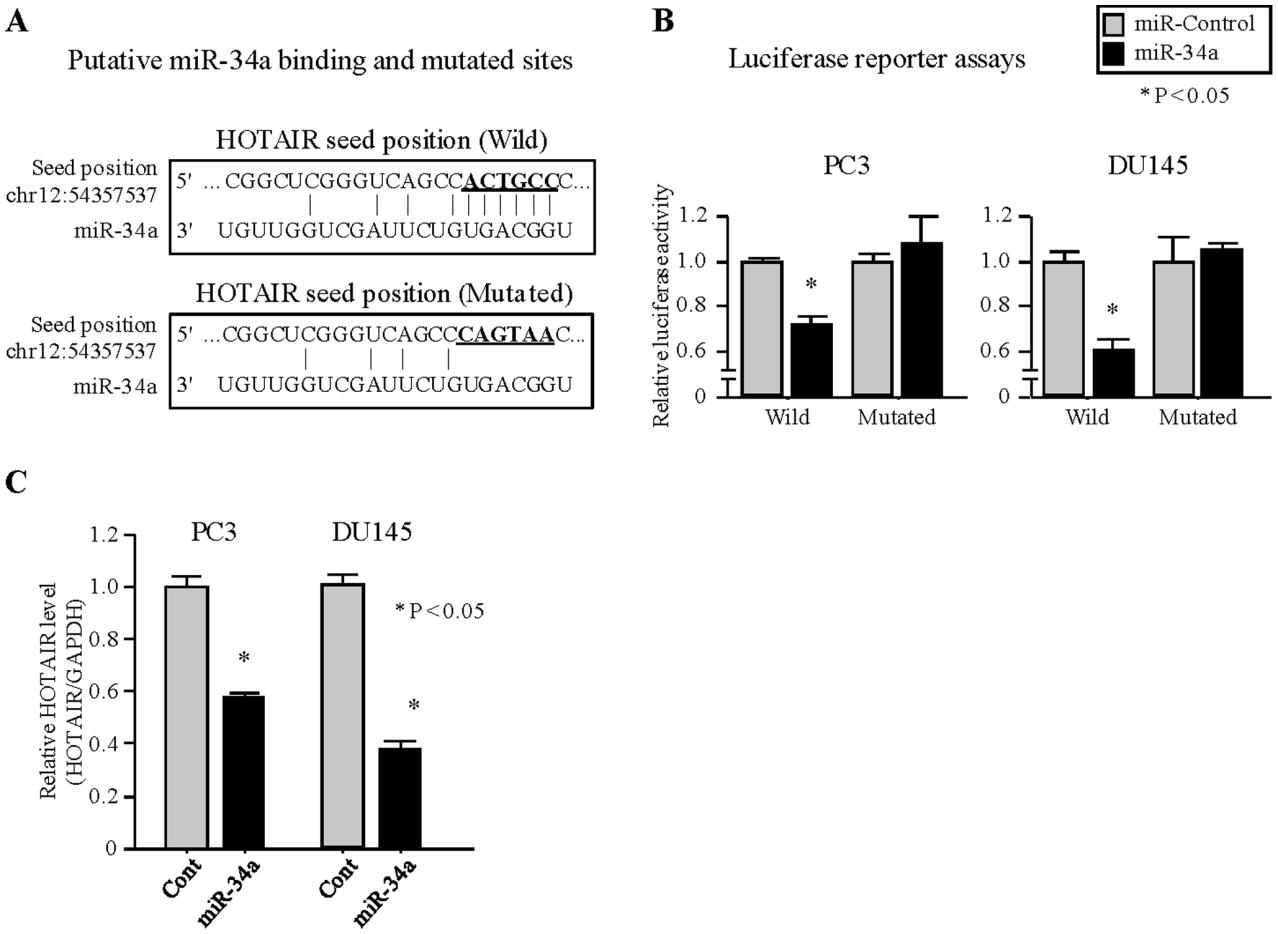
miR-34a regulates HOTAIR expression. (A) Putative miR-34a binding and mutated sites in HOTAIR. (B) Luciferase reporter assays using vectors encoding putative binding sites. PC3 and DU145 cells were transiently transfected with Pre-miR miRNA precursor or negative control, followed by transient transfection with basic vector or wild-type reporter plasmids or mutated plasmids for 24 hours. Reporter activity was measured by luciferase assay and normalized to the activity of Renilla luciferase. Data are presented as the mean ± SE. *, P<0.05. (C) The expression level of HOTAIR was determined by quantitative real-time PCR analyses after transfection with miR-34a mimics and negative control in PCa cell lines (PC3 and DU145). HOTAIR expression was normalized to GAPDH. *, P<0.05.

In order to confirm the binding of miR-34a to HOTAIR, we performed luciferase reporter assays. HOTAIR has one predicted binding site for miR-34a (Fig. 3A) which we cloned into a luciferase reporter assay vector. Luciferase reporter assays demonstrated that miR-34a decreased the relative luciferase activities of the wild type vector (Fig. 3B). Mutation of the putative miR-34a binding sites also decreased the response to miR-34a indicating that miR-34a binds directly to HOTAIR. Quantitative real-time PCR analysis showed that the expression levels of HOTAIR in PC3 and DU145 were repressed in the miR-34a transfectants compared with the controls (Fig. 3C).

### In vitro and in vivo Effect of HOTAIR on PCa Cell Proliferation, Migration, and Invasion

**Figure 4:**
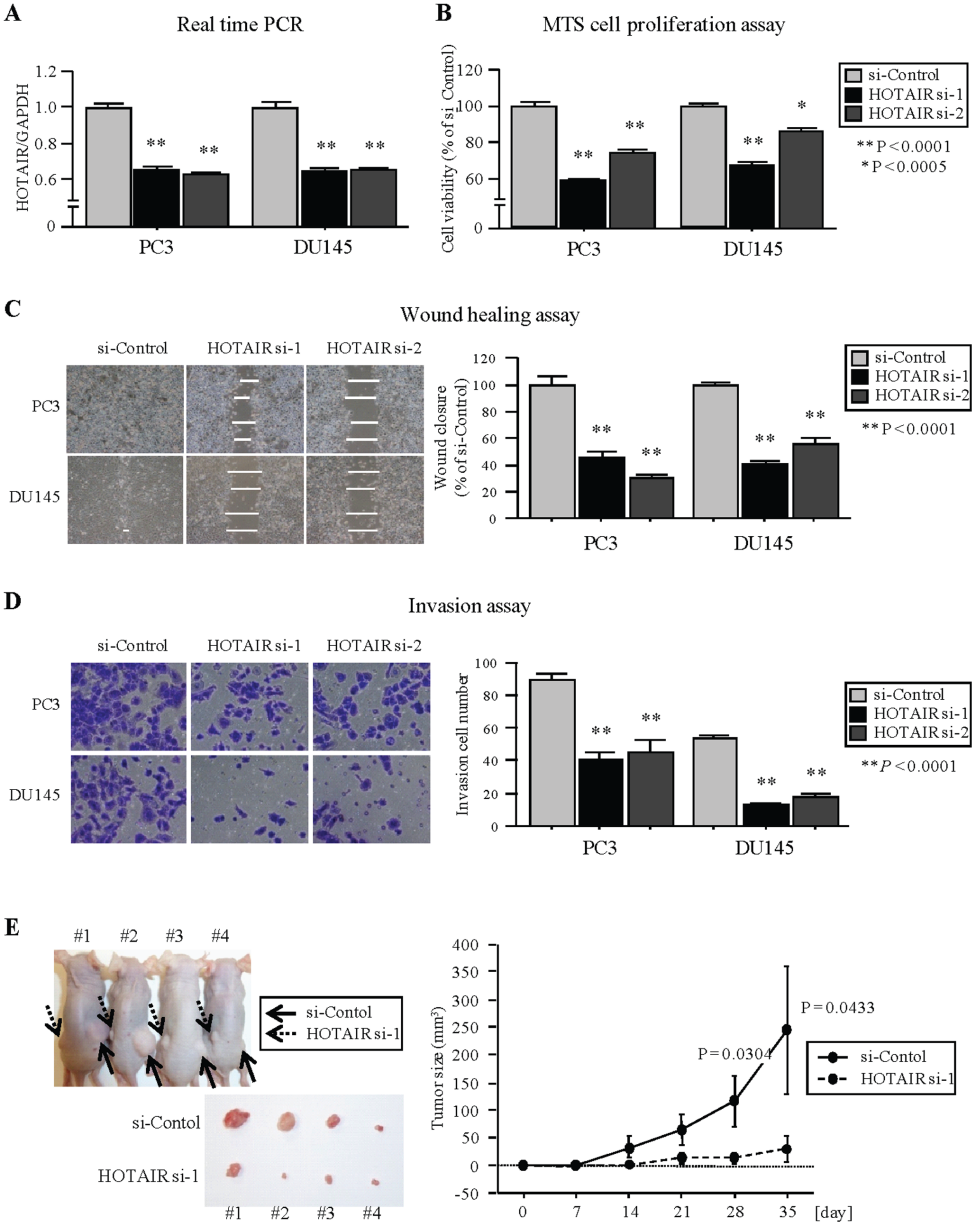
siRNA knockdown of HOTAIR and effect on PCa cell viability. (A) HOTAIR expression levels in PCa cell lines (PC3 and DU145) were determined by real-time PCR at 96 hours after transient transfection of siRNA. Gene expression was normalized to GAPDH. Data are presented as the mean ± SE. **, P<0.0001. *, P<0.0005. (B) Knockdown of HOTAIR significantly inhibits cell viability. Cell viability was analyzed by the MTS cell proliferation assay 96 hours after transient transfection. (C) Knockdown of HOTAIR significantly inhibits cell migration. After transfection (48 hours), a wound was formed by scraping and measured after 24 hours. Representative images of wound healing assay are shown at 200× magnification. **, P<0.0001. (D) Knockdown of HOTAIR significantly decreased cell invasion. Representative images of invasion assay are shown at 200× magnification. **, P<0.0001. (E) Representative images of tumors in nude mice 5 weeks after subcutaneous injection of transfected HOTAIR siRNA DU145 cell lines or control cell lines and time course of tumor growth.

To examine the functional role of HOTAIR, we performed loss-of-function studies using si-RNA knockdown with PC3 and DU145 cells. We initially confined that the expression of HOTAIR was markedly repressed in si-RNA transfectants compared to controls (Fig. 4A). Cell proliferation (Fig. 4B) and wound healing assay (Fig. 4C) showed significant inhibition in si-HOTAIR transfectants in both PC3 and DU145 cells compared to the control transfectants. Invasion assay (Matrigel) also showed that the number of invading cells was significantly decreased in si-HOTAIR transfectants compared with their control counterparts (Fig. 4D). To confirm the effect of HOTAIR on tumorigenicity in vivo, HOTAIR siRNA and si-control-transfected DU145 cells were subcutaneously injected into nude mice. We observed that knock-down of HOTAIR expression inhibited DU145 cell tumor formation in vivo (Fig. 4E). These results suggest that HOTAIR plays an important role in PCa cell progression.

### HOTAIR Influences Cellular Apoptosis and Cell Cycle in PCa Cells

**Figure 5:**
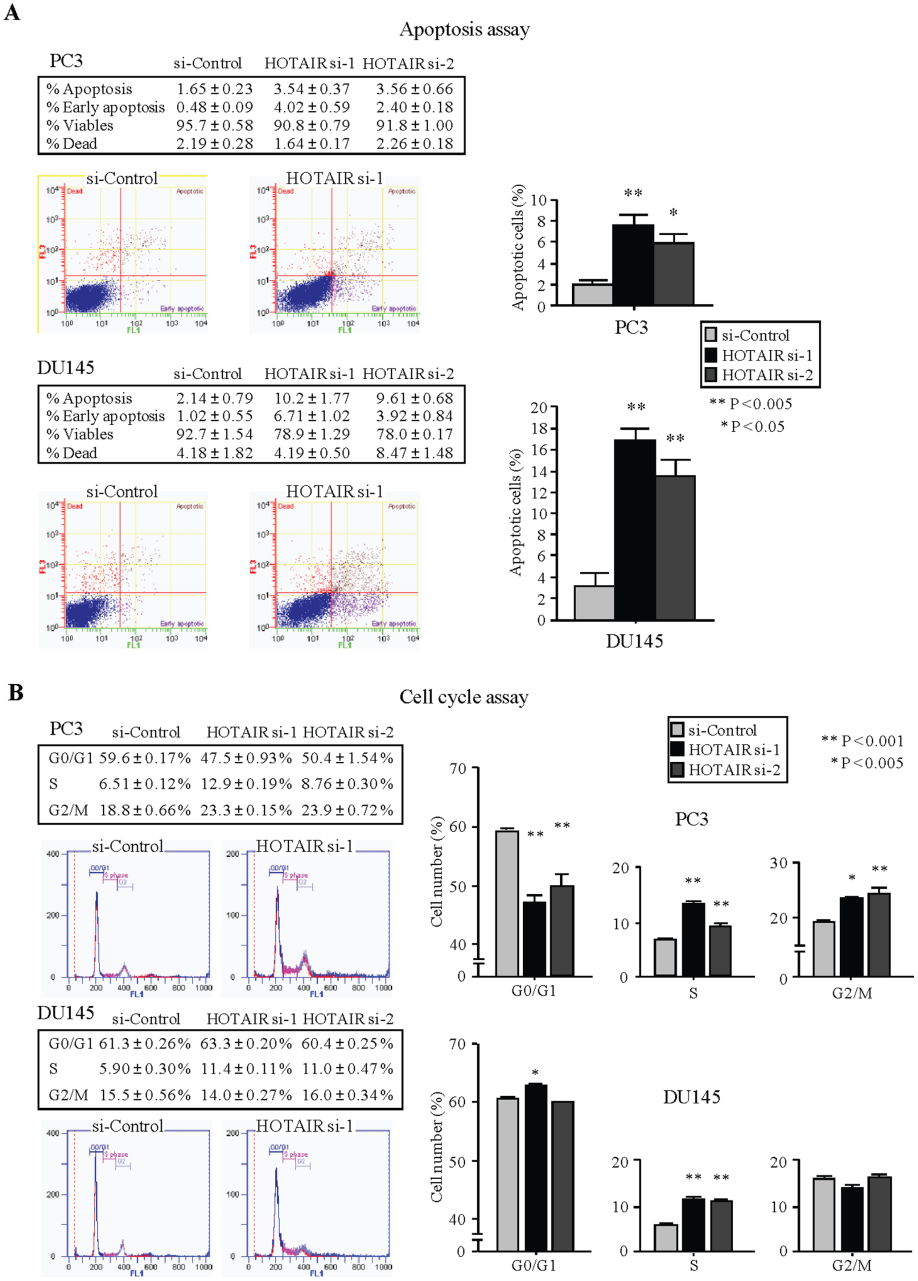
siRNA knockdown of HOTAIR and effect on PCa apoptosis and cell cycle. (A) Apoptosis assay using flow cytometry. Representative quadrant figures of control and si-HOTAIR treated cells in PC3 (upper) and DU145 (lower) cells. **, P<0.005. *, P<0.05. (B) Typical figures of cell cycle analysis of control, or si-HOTAIR treated cells are shown. The bar chart shown on the right of each figure represents the percentage of the cells in G0/G1, S, or G2/M phase as indicated. **, P<0.001. *, P<0.005.

Apoptosis assay demonstrated that knock-down of HOTAIR significantly induced apoptosis in both PC3 and DU145 cells (Fig. 5A). Cell cycle assay showed that knock-down of HOTAIR caused G2/M phase cell cycle arrest in PC3 cells (Fig. 5B) while DU145 cells transfected had a significant increase in the S phase of the cell cycle.

## Discussion

Protein-coding genes comprise only a small part of the genome, and the rest consists of ncRNAs, introns and/or other sequences to which no function has yet been assigned [39]. NcRNAs (miRNAs and lncRNAs) have been shown to play a critical role in regulation of cellular processes such as cell growth and apoptosis in normal and malignant cells. The roles of miRNAs in cancers have been extensively investigated and recently, many lncRNAs have been associated with the development and progression of cancer. The expression of lncRNAs frequently changes during malignant transformation and ncRNAs are emerging as key molecules in human cancer, with the potential to serve as a novel markers and therapeutic targets. However, to date the role of only a few lncRNAs have been characterized in PCa. High-throughput sequencing showed that prostate cancer–associated ncRNA transcript 1 (PCAT-1) is a prostate-specific regulator of cell proliferation in PCa and a target of PRC2 [40]. Prostate cancer gene expression marker 1 (PCGEM1) polymorphisms may contribute to PCa risk [41]. Expression of PlncRNA-1 is significantly higher in PCa and this lncRNA regulates cell proliferation and apoptosis through targeting androgen receptor [42]. In this study, we focused on lncRNAs regulated by genistein in PCa cells and identified HOTAIR in the expression profile of these lincRNAs.

In this study, we found high expression of HOTAIR in castration-resistant PCa cell lines. Our functional assays showed that knockdown of HOTAIR decreased PCa cell proliferation in vitro and in vivo and induced apoptosis. Therefore HOTAIR may play a functional role in tumor cell growth in PCa. Increasing evidence suggests that HOTAIR regulates key pathways in cancer invasion and metastasis. HOTAIR increased breast cancer invasiveness and metastasis by inducing positive regulators of cancer metastasis (ABL2 SNAIL, LAMB3 and LAMC2) in a manner dependent on PRC2 [29]. High HOTAIR expression is a risk for recurrence after hepatectomy in hepatocellular carcinoma and may regulate the MMP9 and VEGF genes [34]. Our functional assays showed that knockdown of HOTAIR decreased PCa cell migration and invasion. Our mRNA array data and bioinformatic analysis also showed that genistein targets MMP9 and VEGF genes that are components of the KEGG ‘Pathway in cancer’. These results indicate that HOTAIR may play an important role in PCa progression.

As gene regulators, miRNAs bind to the 3′UTR of mRNA and can individually target a number of protein-coding genes. However, it remains to be determined whether miRNAs can also target lncRNAs. Recently, a few studies have described the interactions between miRNAs and lncRNAs. miR-29 indirectly regulates expression of tumor suppressor lncRNA, maternally expressed 3 (MEG3) by acting on its methylation in hepatocellular cancer [35]. miR-671 directs cleavage of an antisense transcript of the cerebellar degeneration-related protein 1, 34 kDa (CDR1), leading to a concomitant decrease in CDR1 [37]. In the present study, we looked to see if miR-34a impacts lncRNA expression. Our luciferase reporter assay and real-time PCR results showed that miR-34a binds to the HOTAIR mRNA sequence and down-regulates HOTAIR expression in PCa cell lines. Therefore this study is the first to demonstrate that miR-34a directly targets HOTAIR in both PC3 and DU145 PCa cells.

In conclusion, our results showed that genistein inhibits PCa cell growth through down-regulation of oncogenic HOTAIR that is targeted by tumor suppressor miR-34a. These findings enhance our understanding of how genistein interacts with lncRNA in PCa and indicates additional potential targets for PCa therapy.

## Materials and Methods

### Cell Culture

Human PCa cell lines, LNCaP, PC3 and DU145 and a non-malignant epithelial prostate cell line, RWPE-1, were purchased from The American Type Culture Collection (Manassas, VA, USA). The PCa cell lines were cultured in RPMI 1640 medium supplemented with 10% fetal bovine serum (FBS) in a humidified atmosphere of 5% CO2 and 95% air at 37°C. RWPE-1 cell line was cultured in keratinocyte growth medium supplemented with 5 ng/mL human recombinant epidermal growth factor and 0.05 mg/mL bovine pituitary extract (Invitrogen, Carlsbad, CA, USA). Subconfluent cells (60%–70% confluent) were treated with genistein (25 µmol/L; Sigma, St Louis, MO, USA) and cells treated with vehicle (dimethylsulfoxide) served as control. Cell media and genistein were changed every day and cells were grown for 4 days.

### RNA Extraction

Total RNA was extracted from PCa cell lines and a non-malignant epithelial prostate cell line using a miRNeasy mini kit (Qiagen, Valencia, CA, USA) according to the manufacturer’s instructions.

### Microarray

For microarray, total RNA was extracted from PC3 and DU145 cells treated with genistein using a miRNeasy Mini Kit. SurePrint G3 Human GE 8×60K Microarray (Agilent Technologies, Santa Clara, CA, USA) was used for expression profiling of genistein treated and control cells. The current microarray data were approved by the GEO and were assigned GEO accession number GSE47657.

### Quantitative Real-time PCR

Extracted total RNA was reverse transcribed into single-stranded cDNA using an iScript cDNA Synthesis Kit (Bio-Rad, Hercules, CA, USA) and a TaqMan MicroRNA Reverse Transcription Kit (Applied Biosystems, Foster City, CA, USA). Quantitative real-time PCR analysis was performed with an Applied Biosystems Prism7500 Fast Sequence Detection System using TaqMan universal PCR master mix according to the manufacturer’s protocol (Applied Biosystems). Levels of RNA expression were determined using the 7500 Fast System SDS software version 1.3.1 (Applied Biosystems). PCR parameters for cycling were as follows: 95°C for 20 seconds, 40 cycles of PCR at 95°C for 3 seconds, and 60°C for 30 seconds. All reactions were done in a 10-µL reaction volume in triplicate. The data were analyzed with the delta-delta Ct method to calculate the fold-change. TaqMan probes and primers for HOTAIR (assay ID: Hs03296680_s1), GAPDH (assay ID: Hs02758991_g1), miR-34a (assay ID: 000426), and RNU48 (Assay ID: 001006) were obtained from Applied Biosystems. GAPDH and RNU48 were used as internal controls.

### Transfection

HOTAIR siRNA (SASI_Hs02_00380445 and SASI_Hs02_00380446, Sigma) and negative control siRNA (D-001810-10; Thermo Fisher Scientific, Waltham, MA, USA) were used in loss-of-function experiments. Pre-miR miRNA precursor and negative control (Applied Biosystems) were used in gain-of-function experiments. PC3 and DU145 cells were transiently transfected using Lipofectamine 2000 transfection reagent (Invitrogen), according to the manufacturer’s recommendations.

### Cell Proliferation, Migration, and Invasion Assays

Cell proliferation was measured using a CellTiter 96 AQueous One Solution Cell Proliferation Assay (MTS) kit (Promega, Madison, WI, USA) performed according to the manufacturer’s instructions. Cell proliferation was determined by absorbance measurements at 490 nm using SpectraMAX 190 (Molecular Devices Co., Sunnyvale, CA, USA). Cell migration activity was evaluated by wound-healing assay. Cells were plated in six-well dishes, and the cell monolayers were scraped using a P-20 micropipette tip. The width of the initial gap (0 h) and the residual gap 24 hours after wounding were calculated from photomicrographs. Cell invasion assay was carried out using modified Boyden Chambers consisting of transwell-precoated Matrigel membrane filter inserts with eight micron pores in 24-well tissue culture plates (BD Biosciences, Bedford, MA, USA). Minimum essential medium containing 10% FBS in the lower chamber served as the chemoattractant, as described previously [43]. All experiments were performed in triplicate.

### In vivo Tumor Growth

All animal care was in accordance with the guidelines of the San Francisco Veterans Affairs Medical Center and the study was approved by the San Francisco VA IACUC (Protocol number: 11-008-01). Animal users have completed training programs to handle and work with mice through AALAS (American Association for Laboratory Animal Science) prior to animal experiments. For the subcutaneous xenograft mouse model, DU145 cells (5×10^6^) that were transiently transfected with si-HOTAIR or si-control were suspended in 100 µL RPMI 1640 medium and subcutaneously injected into the left and right backside flanks of female nude mice, respectively. (strain BALB/c nu/nu; Charles River Laboratories, Inc., Wilmington, MA, USA, 5 weeks old). A total of 4 nude mice were used and tumor growth was examined over the course of 35 days. Tumor volume was calculated on the basis of width (x) and length (y): x^2^y/2, where x<y.

### Apoptosis and Cell Cycle Assays

Fluorescence-activated cell-sorting (FACS) analysis for apoptosis was done 96 hours post-transfection, using a Annexin V-FITC/7-AAD Kit (Beckman Coulter, Brea, CA, USA), according to the manufacturer’s protocol. Cell cycle analysis was performed 96 hours after transfection. The cells were harvested, washed with cold PBS and resuspended in the nuclear stain 49,6-diamidino-2-phenylindole (Beckman Coulter). Stained cells were immediately analyzed with a flow cytometer (Cell Lab Quanta SC; Beckman Coulter).

### Identification of Genistein Regulated Target Genes and Bioinformatic Analysis

To search for genes that are regulated by genistein, we performed microarray. To identify the biological processes or pathways potentially regulated by genistein, we performed GeneCodis analysis [44], [45] using all of the candidate genes. Then, to identify the networks among genistein and their target genes, we analyzed and characterized those genes in GO Biological Process and KEGG pathway categories. These data were used to examine genistein-regulated molecular networks in human cells. We performed gene expression analyses of all candidate genes involved in each of the pathways using microarray expression data, which were approved by the GEO and were assigned GEO accession numbers (GSE29079). In the Affymetrix Human Exon 1.0 ST Array (Affymetrix, Santa Clara, CA, USA) datasets, we examined 47 PCa tissues and 47 normal prostate tissues, all of which were collected from patients who had not been exposed to neo-adjuvant radio-, cytotoxic- or endocrine therapy before the operation. The data was normalized and analyzed with the GeneSpring (Agilent Technologies). Statistical analyses were conducted using the Mann Whitney U-test with cut-off P<0.05, and the results are shown as a heat map diagram.

### Plasmid Construction and Dual-luciferase Reporter Assays

To search for miRNA targets of HOTAIR, we used the miRcode algorithm (release 6.2, http://www.mircode.org/). MiRcode provides human miRNA target predictions based on the comprehensive GENCODE gene annotation [46], including >10,000 long non-coding RNA genes. For luciferase reporter assay, PmirGLO Dual-Luciferase miRNA Target Expression Vector was used (Promega). The oligonucleotide sequences (wild-type) used are shown in Table S3. We also constructed mutated oligonucleotides for each of the wild-type oligonucleotides (Table S3). In a total volume of 25 µl, 1 µl each of 100 µM forward and reverse oligonucleotide, 2.5 µl of 10 X annealing buffer (100 mM Tris–HCl, pH 7.5, 1 M NaCl and 10 mM ethylenediaminetetraacetic acid) and 20.5 µl water were incubated at 95°C for 3 min and then placed at 37°C for 15 min. The oligonucleotides were ligated into the PmeI–XbaI site of pmirGLO Dual-Luciferase miRNA Target Expression Vector. For luciferase reporter assay, PCa cells were co-transfected with Pre-miR miRNA precursor and pmirGLO Dual-Luciferase miRNA Target Expression Vectors using Lipofectamine 2000 (Invitrogen) and X-tremeGENE HP DNA Transfection Reagent (Roche Diagnosis, Basel, Switzerland, USA) according to the manufacturer’s instructions. Luciferase reporter assay was performed using the Dual-Luciferase Reporter Assay System (Promega) 24 hours after transfection.

### Statistical Analysis

The relationship between two variables and the numerical values obtained by real-time RT-PCR were analyzed using the nonparametric Mann-Whitney U test. All analyses were performed using Expert StatView (version 4, SAS Institute Inc.). Data are shown as mean values ± standard error. In the comparison among three variables, a nonadjusted statistical level of significance of P<0.05 corresponds to a Bonferroni-adjusted level of P<0.0167.

## Supporting Information

Figure S1 Putative genistein regulated genes in ‘Pathway in cancer’. The putative genistein regulated genes (highlighted in red) as defined by Kyoto Encyclopedia of Genes and Genomes (KEGG) pathway and determined through GENECODIS analysis. (TIF)

Figure S2 The heat map diagram of the “Pathways in cancer”. Analysis show prostate cancer tissues (n = 47) and normal prostate samples (n = 47). Each square represents the expression level of a given gene in an individual sample. Red represents increased expression and blue represents decreased expression relative to the normalised expression of the gene across all samples. (TIF)

Table S1 Genes downregulated by genistein. (XLS)

Table S2 Pathways regulated by the targets of genistein (KEGG annotation). (XLS)

Table S3 Primer oligonucleotide sequences (wild-type and mutated). (XLS)
