# Supplementary figures and images for: Genistein Inhibits Prostate Cancer Cell Growth by Targeting miR-34a and Oncogenic HOTAIR
Source: PLoS One. 2013 Aug 1;8(8):e70372. doi: 10.1371/journal.pone.0070372 (PMC3731248; doi:10.1371/journal.pone.0070372)

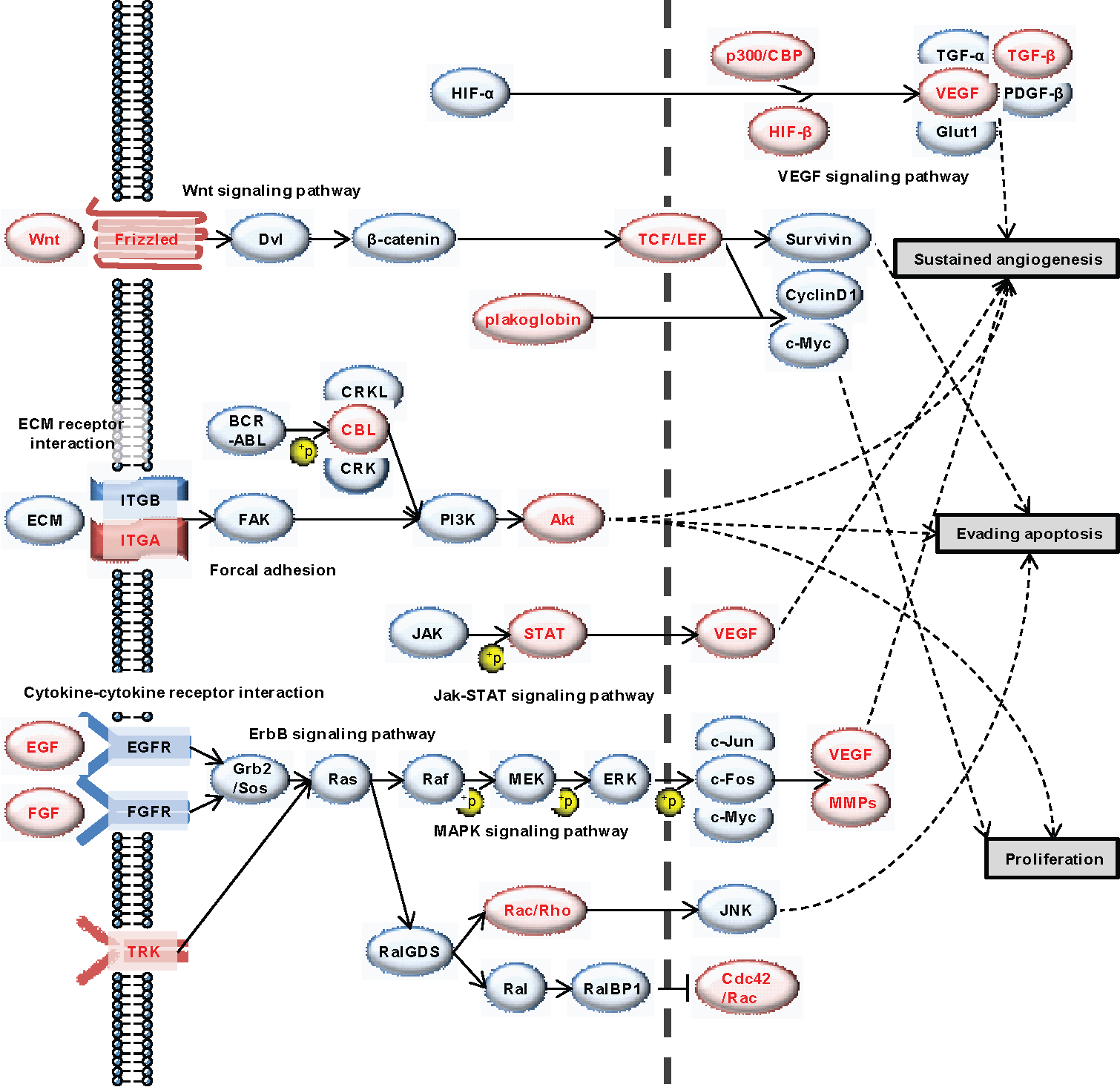

Supplement: Figure S1 — (TIF) [file pone.0070372.s001.tif]

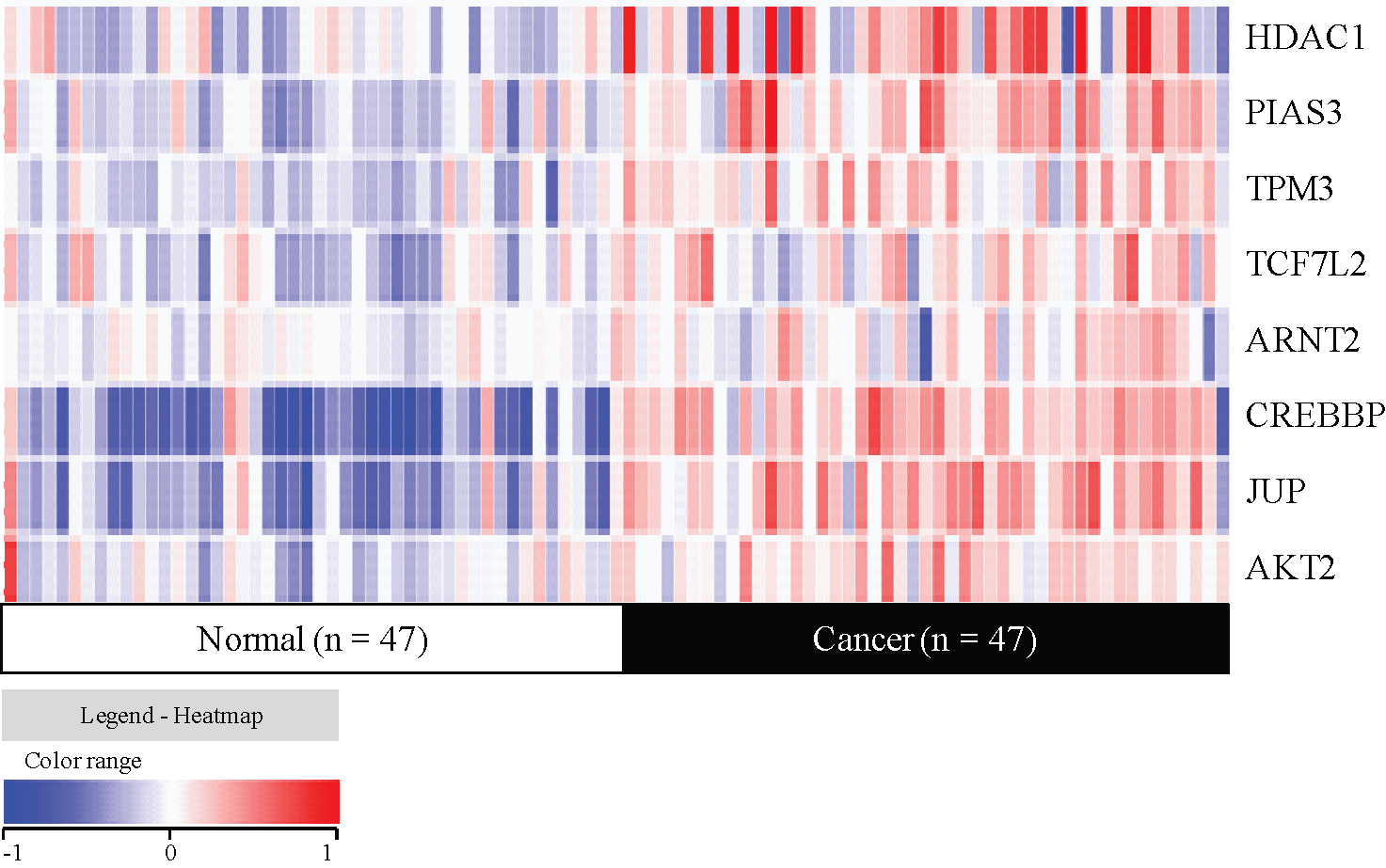

Supplement: Figure S2 — (TIF) [file pone.0070372.s002.tif]
